# Temporal Trends in Internal vs. External Referrals for TAVR in a Large Academic Center: Patients Characteristics and Outcomes

**DOI:** 10.1155/2022/6074368

**Published:** 2022-08-05

**Authors:** Aurelie Merlo, Audrey Khoury, Mohsin Shah, Tabitha Linville, John Ikonomidis, Matthew Cavender, John Vavalle, Thomas Caranasos

**Affiliations:** ^1^Department of Surgery, Division of Cardiothoracic Surgery, University of North Carolina, Chapel Hill, North Carolina, USA; ^2^Department of Medicine, Division of Cardiology, University of North Carolina, Chapel Hill, North Carolina, USA

## Abstract

**Background:**

Since transcatheter aortic valve replacement (TAVR) first became approved for inoperable patients followed by high, intermediate-, and low-risk patients, referrals to TAVR centers have rapidly increased. The purpose of this study was to investigate referral patterns to a large academic TAVR center in the state of North Carolina and evaluate differences between externally and internally referred patients.

**Methods:**

Data for all patients who underwent TAVR at our institution between November 2014 and March 2020 were pulled from the Transcatheter Valve Therapy Registry. The electronic medical record was used to determine the referral source. The descriptive statistical analysis was performed using Excel (Microsoft, Redmond, Washington).

**Results:**

491 patients underwent TAVR at our institution between November 2014 and March 2020. Half of the patients were referred by a cardiologist within the same health system (*N* = 250, 50.9%). Other referral sources included a cardiologist external to the health system (*N* = 210, *N* = 42.8%) and a surgeon or proceduralist (such as urologist, surgeon, or gastroenterologist) during the workup for another procedure (*N* = 26, 5.3%). Over time, there was a trend toward an increasing proportion of patients referred by a cardiologist external to our system, but this trend did not reach statistical significance (20.0% in 2014, 29.2% in 2015, 30.7% in 2016, 53.0% in 2017, 36% in 2018, 48.4% in 2019, and 56.8% in 2020, *p*=0.06 using the Mann–Kendall trend test). Externally referred patients were less likely to have private insurance and were more likely to have a reduced ejection fraction and had a higher mean gradient across the valve. Postprocedure, externally referred patients were more likely to have the procedure under moderate sedation and less likely to be discharged home.

**Conclusions:**

This study presents the referral pattern to a large TAVR center in North Carolina. Over time, there was an increase in external referrals suggesting that TAVR is increasingly adopted as an important component of the management of aortic valve stenosis. Internally and externally referred patients have differences in baseline demographic and clinical characteristics which may have an impact on clinical outcomes.

## 1. Introduction

The advent of transcatheter technologies has revolutionized the field of aortic valve surgery, initially in high-risk patients and now extending in lower-risk patient populations. Transcatheter aortic valve replacement (TAVR) was first described in 2002 for a patient with critical aortic stenosis who had been declared inoperable [1], and yet the patient experienced an excellent result. This first in human experience sparked a rapid investigation into and development of TAVR technology. Since the initial trials, the indication for TAVR has expanded from inoperable patients and high-risk patients with the PARTNER trial in 2010 [2], to intermediate-risk patients with the PARNER2 and SURTAVI trials in 2016–2017 [3, 4], and finally to low-risk patients with the PARTNER3 and EVOLUT trials in 2019 (5, 6). One of the reasons for this expeditious evolution of technology is due to the remarkable outcomes that TAVR valves can offer. The latest trials in low-risk patients demonstrate freedom from the composite endpoint of death/stroke/rehospitalization of 91% [5] at one year and freedom from death/disabling stroke of 94% at one year [6]. Most modern series describe a length of stay of two to six days [7–9].

With the aforementioned outcomes and expansion of approval for TAVR in low-risk patients, heart centers across the country now offer TAVR as part of the armamentarium for addressing severe aortic stenosis. Within this context, referrals to TAVR centers have also increased [10], and in some countries, such as Canada, the surge in referrals has resulted in long waitlists to undergo the procedure [11]. Unfortunately, referral patterns to large TAVR centers are not well studied or understood. Understanding these referral patterns is critical for understanding factors related to program growth and addressing areas for targeted improvement in program outreach. The purpose of this study was to define referral patterns to a large academic TAVR center in the state of North Carolina and determine if there were demographic or clinical differences between internally referred and externally referred patients.

## 2. Methods

All patients who underwent TAVR at the University of North Carolina (UNC) between November 2014 and March 2020 were included in this study. All TAVR patients were entered into our institution's Transcatheter Valve Therapy (TVT) Registry database. This datafile was extracted from the online database and we performed a retrospective chart review of all patients to determine the referral source. The referral source was categorized as internal cardiologist, external cardiologist, procedure, self, or unknown. External cardiologist was defined as a cardiologist who does not work within the UNC hospital system. Procedure referral was defined as any patient who was referred for TAVR evaluation during the workup for another procedure, such as kidney transplant, colonoscopy, or urethral stent placement.

All data extraction was conducted within the standard of our hospital's Institutional Review Board Committee (Study number 20-0606). The requirement for consent from individual study participants was waived given the retrospective nature of this chart review. Excel (Microsoft, Redmond, Washington) was used to perform descriptive and comparative statistical analysis, and the Mann–Kendall test was used to analyze temporal trends. *T*-tests and proportion tests were used to compare externally and internally referred patients. A *p* value < 0.05 was considered statistically significant.

## 3. Results

### 3.1. Study Population

491 patients underwent TAVR at our institution between November 2014 and March 2020. Baseline characteristics (Table 1) of the study population are noted in Table 1. The median patient age was 80.5 years, and the majority of patients were white (81.1%) and privately insured (66.2%). The most common comorbidities were hypertension (89.2%), atrial fibrillation (34.0%), and diabetes (39.1%). The average STS risk score was 5.5, 76% of patients had New York Heart Association Class III and IV, and 16% of patients had an ejection fraction less than 40%. 4% of patients had bicuspid aortic valves and the mean annulus size was 24.6 mm, the average valve area was 0.74 cm^2^, and the mean gradient across the valve was 41.8 mmHg. Externally referred patients were less likely to have insurance, but the remainder of the demographic factors were similar between groups. Externally referred patients were also more likely to have a reduced ejection fraction and a higher peak gradient across the aortic valve preprocedure.

### 3.2. Procedure/Patient Characteristics

The majority of TAVR implantations were performed with moderate sedation (60.9%) and 6.1% were valve-in-valve implantations (Table 2). Postimplantation hemodynamics included an average postprocedure gradient of 5.4 mmHg, aortic valve area of 1.9 cm^2^, and maximum velocity of 2.2 m^2^; only 2% of patients had moderate or severe aortic regurgitation postprocedure. 98% of patients survived to discharge and 86% of patients were discharged home.

Patients who were externally referred were more likely to have the procedure performed under moderate sedation (rather than general anesthesia), but they were less likely to be discharged home. The remainder of the postprocedure hemodynamic parameters were similar between groups.

### 3.3. Referral Pattern

**Figure 1:**
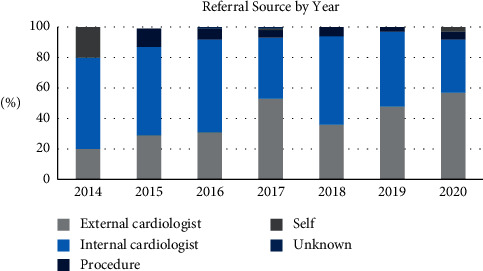
Referral source by year for all TAVRs performed at a single institution.

Approximately half of the patients were referred by a cardiologist within the same health system (*N* = 250, 50.9%) (Table 3). Other referral sources included a cardiologist external to the health system (*N* = 210, *N* = 42.8%) or a proceduralist during the workup for another procedure (*N* = 26, 5.3%). Three patients were self-referred and two patients had an unknown source of referral. Over time, there seemed to be a trend toward a higher proportion of referrals being made by cardiologists external to our system, but this trend was not statistically significant (20.0% in 2014, 29.2% in 2015, 30.7% in 2016, 53.0% in 2017, 36% in 2018, 48.4% in 2019, and 56.8% in 2020, *p*=0.06 using Mann–Kendall trend test) (Figure 1). One explanation for the decrease in referrals in 2018 is that hospital systems underwent a merger impacting internal and external referrals during that year, causing a reclassification of internal and external referring providers. When we repeated the Mann–Kendall test excluding the data from 2018, the trend toward an increase in external referrals was significant (*p*=0.024).

### 3.4. Referral during Workup for Another Procedure

Twenty-six patients (5.3%) were referred during the workup to undergo another procedure. The details regarding that referral process are included in Table 4. Nearly half of these patients were able to undergo their initial procedure within a year of undergoing TAVR (*N* = 12, 46.2%) (Table 4).

## 4. Discussion

This study describes the referral patterns at a large academic TAVR center. Notable findings include that approximately half of all referrals originated from a cardiologist within the same hospital system and five percent of referrals came from a proceduralist. The ratio of referrals from internal versus external cardiologists has remained relatively stable over time, with a nonstatistically significant trend toward an increase in referrals from external cardiologists over time. We did see a statistically significant trend when excluding the data from the year 2018 during which the hospital system was undergoing a merger with another TAVR center. These findings may indicate a trend toward increased adoption of TAVR technology in the community.

In addition, this study demonstrates that externally referred patients are less likely to have insurance. This is consistent with referrals made to public hospitals. Notably externally referred patients were also more likely to have a reduced ejection fraction and a higher peak gradient. This may be related to different standards within the cardiologists at our institution and in the community regarding the timing of referral to TAVR, or may demonstrate a later presentation to care in this population that was less likely to have a private insurance.

The impact of the differences in presentation on clinical outcomes is unclear. The fact is that more externally referred patients who underwent the procedure with moderate sedation may be related to the increase in external referrals over time or may be related to the higher proportion of these patients having a reduced ejection fraction preoperatively. It is likely that the reduced proportion of patients discharged home who were referred externally is related to the reduced number of private insurance and a sicker presentation, but the exact causal mechanism is not clear.

The findings of this study have been replicated in other studies. An analysis by Buchanan et al. demonstrated that the rate of TAVR exclusion has decreased by half over a six-year period in their institution [10]. An analysis of the Healthcare Cost and Utilization Project state inpatient databases in the states of Arizona, Florida, Iowa, Massachusetts, and Washington may suggest a mechanism as the authors demonstrated that patients treated at high-competition hospitals had higher odds of receiving TAVR, relative to patients at low-competition hospitals [12].

The majority of TAVR patients at our institution were white and privately insured patients. The gender breakdown was equal in our cohort, which is becoming more common in recent single institution TAVR studies [13, 14] (in contrast to the male predominance noted in early studies [15]). The most common comorbidities were hypertension, diabetes, and atrial fibrillation, which are again comorbidities frequently seen in other studies. While the majority of the patients in this cohort were white (80%), this is lower than the white predominance of 90% frequently reported at other centers [12, 13] if reported at all [16].

These referral patterns need to be implemented within the healthcare environment of North Carolina. The state includes sixteen TAVR centers (Table 5), many of which have opened in the past two to three years. Other contributors include market trajectories that are difficult to identify, such as hospital mergers and changes in referral groups.

A particular interest of our study was to consider patients who were referred for TAVR during the workup for another procedure, and we were interested in discovering if these patients underwent that procedure within the first year after TAVR. We did note that 5.3% of referrals were from proceduralists, and close to half of these patients (46.2%) underwent their referral procedure within a year after TAVR. Furthermore, two of those patients were listed for kidney transplant within a year even if they did not undergo a transplant, and another patient elected to not have surgery. This study provides further evidence of the benefit to patients of the minimally invasive nature of TAVR and the shorter recovery time, allowing patients to undergo procedures within a year after TAVR.

### 4.1. Limitations

This study contains all of the limitations of a large retrospective chart review, even with the majority of the data collection (regarding patient and procedure characteristics) being performed by a dedicated clinical research coordinator using well-established definitions put forth via the Society of Thoracic Surgeons/American College of Cardiology (STS/ACC) TVT Registry. The additional data collection for referral patterns was simple and involved determining the referring source. Additionally, this study was not powered to analyze temporal trends in referral patterns. It is important to note that, since data collection for the years 2014 and 2020 were not full years, raw numbers could not be used for the analysis.

Unfortunately, the time from when the referral was placed to the time of the procedure was not available with our dataset. It is possible that a difference in timing of workup and timing of procedure between externally referred patients and internally referred patients is contributing to the differences in outcomes. If there is a referral made to the heart valve center, all needed studies are scheduled the same day. It is rare that patients need an additional visit prior to deciding whether TAVR is needed. Nonetheless, delays in procedure timing or in referring patients are not accounted for in this analysis.

Finally, additional data from other imaging modalities such as computed tomography calcium scoring and dobutamine stress echocardiography were not collected in this dataset. These are valuable assets in the evaluation of patients with severe aortic stenosis. Given that this information was unavailable for all patients, its availability would likely not impact the differences between externally and internally referred patients but could provide additional insight into the clinical outcomes of these patients, especially when compared to other institutions.

## 5. Conclusion

In summary, this article describes referral patterns at a large academic TAVR center and demonstrates key differences between externally and internally referred patients. In order to remain relevant in a state with a rapid growth of TAVR centers, targeted areas for this center to expand include outreach efforts to increase referrals from cardiologists outside of the health system, and also attempting to receive referrals for these patients earlier in the disease process. Furthermore, increasing internal referrals through referrals from proceduralists who are working up patients for other procedures may be another area to focus on. Finally, our center, a quaternary care hospital, may have a lower percentage of self-referrals, when compared to a community-based program.

## Figures and Tables

**Table 1:** Patient characteristics of 491 consecutive patients undergoing TAVR at a single institution.

	All	Internal	External	Others	*p* value
	N/%	250	210	31	
Age (years)	80.5	81	80	73	0.51
Male	51%	52%	53%	29%	0.90
White	81%	80%	81%	84%	0.89
Black	15%	15%	16%	13%	0.87
Private insurance	66%	73%	60%	55%	<0.01
Peripheral arterial disease	20.4%	20%	21%	16%	0.88
Smoker	9.8%	8%	10%	13%	0.56
Hypertension	89.2%	88%	91%	87%	0.34
Diabetes	39.1%	38%	40%	39%	0.73
Dialysis	4.5%	4%	3%	16%	0.74
Home oxygen	7.9%	6%	10%	6%	0.16
Hostile chest	2.9%	2%	3%	6%	0.70
Immunosuppression	4.9%	6%	3%	6%	0.18
Prior myocardial infarction	13.0%	12%	15%	9%	0.42
Porcelain aorta	2.6%	4%	1%	3%	0.07
Atrial fibrillation/flutter	34.0%	34%	34%	26%	1.00
STS Risk score	5.5	5.7	5.4	4.2	0.53
Body mass index	28.8	26.4	27	27	0.38
NYHA class III + IV	76%	76%	76%	66%	1.00
Preprocedure hemoglobin	12.0	12.1	12.1	11.8	0.74
Preprocedure creatinine	1.08	1.03	1.04	0.8	0.19
% Ejection fraction <40%	16%	0%	20%	3%	<0.01
Moderate or severe aortic insufficiency	17%	18%	15%	20%	0.45
Bicuspid aortic valve	4%	5%	3%	3%	0.34
Peak velocity (meter/second)	4.06	4.01	4.09	4.27	0.29
Annulus size (mm)	24.6	24.6	24.6	23.8	1.00
Aortic valve area (cm^2^)	0.74	0.74	0.73	0.81	0.52
Aortic valve gradient (peak)	41.8	40.5	43.1	43.3	0.03

The *p* value is in reference to comparison between internal and external referrals.

**Table 2:** Procedural outcomes of 491 consecutive patients undergoing TAVR.

	All	Internal	External	Others	*p* value
Valve in valve	6%	6%	6%	3%	1.00
Moderate sedation	61%	56%	66%	56%	0.03
Postmean gradient (mmHg)	5.4	5.2	5.5	6.2	0.80
Post-AVA (cm^2^)	1.9	1.9	1.9	1.7	0.79
Postmaximum velocity (m^2^)	2.2	2.2	2.2	2.3	0.40
Moderate or severe aortic insufficiency	2%	2%	2%	7%	1.00
Alive discharge	98%	99%	97%	100%	0.24
Discharge home	86%	89%	80%	100%	0.01

The *p* value is in reference to comparison between internal and external referrals.

**Table 3:** Referral patterns for 491 patients undergoing TAVR at a single institution.

	2014		2015		2016		2017		2018		2019		2020		Total
External cardiologist	1	20.0%	7	29.2%	23	30.7%	53	53.0%	46	36.0%	59	48.4%	21	56.8%	210
Internal cardiologist	3	60.0%	14	58.3%	46	61.3%	40	40.0%	74	57.8%	60	49.2%	13	35.1%	250
Procedure	0	0.0%	3	12.5%	5	6.7%	5	5.0%	8	6.3%	3	2.5%	2	5.4%	26
Self	1	20.0%	0	0.0%	0	0.0%	1	1.0%	0	0.0%	0	0.0%	1	2.7%	3
Unknown	0	0.0%	0	0.0%	1	1.3%	1	1.0%	0	0.0%	0	0.0%	0	0.0%	2
Total	5		24		75		100		128		122		37		491

**Table 4:** Subset of patients referred by proceduralists and procedural outcomes.

Referral procedure	Underwent referral procedure	Underwent referral procedure within 1 year	Comment
Surgery for endometrial cancer	Yes	Yes	
Melanoma excision	Yes	Yes	
Ureteral stent placement	Yes	Yes	
Kidney transplant	No	No	Had kidney transplant >1 year after referral
Gastric outlet obstruction requiring surgery	Yes	Yes	EGD
Inguinal hernia	Unknown		Lost to follow-up
Lung resection vs. SBRT	Yes	Yes	SBRT
Colonoscopy	Unknown		Lost to follow-up
Chemotherapy and radiation for lung cancer	Yes		
Kidney transplant	No		Listed for kidney transplant
Shoulder surgery	No		
Whipple	No		Patient elected to not have surgery
Parastomal hernia	yes	Yes	
Cholecystectomy	Yes	Yes	
Kidney transplant	Yes	Yes	
Removal breast implant	Yes	Yes	
Ventral hernia repair	Yes	Yes	
Nephrectomy	Yes	No	
Colonoscopy	Yes	No	
Parastomal hernia	Yes	Yes	
Partial nephrectomy	Yes	No	
Ovarian mass	No		
Upper endoscopy	No		
Colovesicular fistula takedown	No		Required DAPT that could not be discontinued
FEVAR	Yes	Yes	
Liver transplant	No		Has not been a year

DAPT, dual antiplatelet therapy; EGD, esophagogastroduodenoscopy; FEVAR, fenestrated endovascular aortic repair; SBRT, stereotactic body radiation therapy.

**Table 5:** List of TAVR centers in North Carolina.

Hospital name	Location	County	Beds	Trauma designation	TAVR
Atrium Health Cabarrus	Concord	Cabarrus	447	Level III	Yes
Atrium Health Carolinas Medical Center	Charlotte	Mecklenburg	874	Level I	Yes
Cape Fear Valley Medical Center	Fayetteville	Cumberland	600	Level III	Yes
CarolinaEast Medical Center	New Bern	Craven	350		Yes
Cone Health Moses Cone Hospital	Greensboro	Guilford	536	Level II	Yes
Duke University Hospital	Durham	Durham	943	Level I	Yes
FirstHealth Moore Regional Hospital	Pinehurst	Moore	402		Yes
Mission Hospital	Asheville	Buncombe	763	Level II	Yes
New Hanover Regional Medical Center	Wilmington	New Hanover	798	Level II	Yes
Novant Health Forsyth Medical Center	Winston-Salem	Forsyth	921		Yes
Novant Health Presbyterian Medical Center	Charlotte	Mecklenburg	622	Level III	Yes
UNC Medical Center	Chapel Hill	Orange	778	Level I	Yes
UNC REX Hospital	Raleigh	Wake	665		Yes
Vidant Medical Center	Greenville	Pitt	974	Level I	Yes
Wake Forest Baptist Medical Center	Winston-Salem	Forsyth	885	Level I	Yes
WakeMed	Raleigh	Wake	567	Level I	Yes

## Data Availability

The majority of the data are held in the TVT registry. Referring physician data are available upon request. Contact Aurelie Merlo at aurelie.merlo@unchealth.unc.edu for additional data access.
